# Detecting early signs of Alzheimer’s disease and related dementia onset from the EHR

**DOI:** 10.1093/geroni/igab046.2441

**Published:** 2021-12-17

**Authors:** Karen Schliep, Zachary Shepelak, Nicolas Bitter, Ramkiran Gouripeddi, Truls Ostbye, Ken Smith, Samir Abdelrahman, Joanne Tschanz

**Affiliations:** 1 University of Utah, Salt Lake City, Utah, United States; 2 Duke University, Durham, North Carolina, United States; 3 Utah State University, Logan, Utah, United States

## Abstract

As dementia is widely under-detected, a predictive model using electronic health records (EHR) could provide a method for early screening to implement preventive strategies. There is limited research on using EHR to identify persons with Alzheimer’s disease (AD) and related dementias (RD). In a data-driven approach, we used all ICD-9 diagnosis and CPT procedure codes from statewide inpatient, ambulatory surgery, and Medicare records, in addition to age at baseline and gender, to detect AD/RD from the Cache County Study on Memory in Aging (1995–2009). After removing participants diagnosed with dementia at baseline (n=335), 3882 (82%) Cache County Study participants could be linked to inpatient, ambulatory surgery, and/or Medicare EHR records; 484 (12.5%) of these 3882 had incident all-cause dementia, with 308 (7.9%) having AD/AD comorbid with RD; and 176 (4.5%) having RD without AD. We removed participant’s ICD-9 codes occurring after first AD/RD diagnoses. EHR features (~2000) along with gold-standard diagnoses as class labels were then used to train and detect AD and/or RD using a Gradient Boosting Trees machine learning algorithm. Models evaluated with nested cross-validation yielded AUCs of 0.70 for all-cause dementia, 0.69 for AD/AD comorbid with RD, and 0.67 for RD without AD. Key factors detecting AD/RD included age at enrollment, cardiovascular, metabolic, and kidney disease, and sleep disturbances, with feature importance varying by record type and time frame prior to dementia onset. Our findings suggest that a patient’s health status up to 12 years prior may be useful in identifying individuals at-risk for dementia development.

